# Long-term functional, anatomical outcome, and qualitative analysis by OCTA, as a predictor of disease recurrences in patients with choroidal neovascularization secondary to angioid streaks

**DOI:** 10.1186/s40942-024-00568-y

**Published:** 2024-07-29

**Authors:** Raul Velez-Montoya, Hillary K. Osorio-Landa, K. Carolina Franco-Ramirez, Victor Martínez-Pacheco, J. Abel Ramirez-Estudillo, Jaime Francisco Rosales-Padrón, Gerardo Ledesma-Gil, Jans Fromow-Guerra

**Affiliations:** 1 Retina Department, Asociación para Evitar le Ceguera en México IAP, #46 Vicente García Torres Street, San Lucas Coyacan, Mexico City, 04030 Mexico; 2 Retina Department, Hospital de Nuestra Señora de la Luz IAP, Ezequiel Montes #135, Tabacalera, Mexico City, 06030 Mexico; 3 Retina Department, Instituto Fundacion Conde de Valenciana, Chimalpopoca #14, Centro, Mexico City, 06800 Mexico

**Keywords:** Angioid streaks, Choroidal neovascularization, Recurrence, Treatment, Optical coherence tomography angiography, Odds

## Abstract

**Background:**

To report the risk of exudation recurrence and long-term outcomes in patients with choroidal neovascularization secondary to angioid streaks, according to its morphology and characteristics by optical coherence tomography angiography.

**Methods:**

Retrospective analysis of electronic medical records from three hospitals. We enrolled patients with a clinical diagnosis of angioid streaks choroidal neovascularization that had a minimum follow-up of 12 months. From each record, we extracted general demographic data, best corrected visual acuity (baseline, before and after each disease recurrence and last on file), type of treatment, time between last intravitreal injection and disease recurrence, and classification of the neovascular lesion morphology by optical coherence tomography, and optical coherence tomography angiography. Patients with myopic choroidal neovascularization were used as controls. Interobserver agreement was assessed with a Cohen-Kappa test. The Odds ratio was calculated with a chi2 test for significance. Visual acuity change through time was evaluated with an ANOVA for repeated measurements with an alpha value of 0.05 for statistical significance.

**Results:**

We enrolled 30 patients in the study group and 14 in the control group. In the study group, the baseline and final BCVA were 0.861 ± 0.59 and 1.095 ± 0.61 logMAR (*p* = 0.1) respectively. Control group: 1.045 ± 0.57 and 0.617 ± 0.53 logMAR (*p* < 0.05). In the study group, the predominant CNV type by OCTA was mixed (37%), and interlacing (57%) in the control group. Mixed and cog-wheel patterns at baseline had increased Odds for recurrence in the study group (*p* = 0.09). Patients in the study group required more intravitreal injections on each recurrence episode to achieve disease control (3.5 ± 1.5 vs.1.4 ± 0.2, *p* < 0.01).

**Conclusions:**

The benefits of anti-VEGF treatment are lost over time in patients with angioid streaks and CNV. Lesion characteristics by optical coherence tomography angiography could help physicians predict the risk of recurrence.

**Trial registration:**

Retrospective registered, and IRB approved.

## Background

Angioid streaks (ASs) are well-defined, bilateral, irregular, brownish-red to light-gray line-like lesions, that originate from the optic nerve, and radiate toward the retinal periphery [1, 2]. Histologically they represent breaks on a debilitated Bruch’s membrane due to a thickened and calcified elastic fiber layer [2, 3] Although they may coexist with several systemic diseases with various degrees of ocular involvement such as Marfan syndrome, Paget disease, sickle cell disease, acromegaly, hemochromatosis, and *pseudoxanthoma elasticum* among others, [3, 4] ASs are usually benign findings in the posterior pole and cause no visual impairment by themselves. However, ASs are frequently complicated by choroidal neovascularization (CNV) in up to 86% of cases, which constitutes the major cause of severe visual impairment in patients with Ass [5].

Morphologically, AS-associated CNV (AS-CNV) resembles lesions observed in myopic CNV more closely than those found in age-related macular degeneration (AMD) [1, 5, 6] On structural optical coherence tomography (OCT), AS-CNV usually appears as Type-2 lesions that cause exudation, hemorrhage, and subsequent subretinal fibrosis in middle-aged working patients [1, 5–8] Their initial response to intravitreal treatment with anti-vascular endothelial growth factor (VEGF) drugs also resembles the response observed in myopic CNV patients more than that observed in AMD patients, with resolution of exudations and significant visual recovery after the first round of treatment [1, 6, 9, 10]. Nevertheless, real-life studies have shown that, in contrast to myopic CNV, AS-CNV is characterized by a high level of recurrence and longer exudative periods, leading to the progressive loss of initial gains even to the point of returning to baseline and, in time, to be more prone to subretinal scarring [1, 5, 11, 12]. It is believed that the main reason for the latter complication could be that anti-VEGF treatment in AS patients is usually reactive rather than proactive [13] This means that patients are more often allocated to a *pro-re-nata* (PRN) treatment regimen that might leave them exposed to future episodes of exudation, which we cannot predict accurately [1, 10, 13].

Optical coherence tomography angiography (OCTA) is a relatively novel imaging technique based on the split-spectrum amplitude decorrelation algorithm that uses the dynamic motion of erythrocytes to produce noninvasive high-resolution en face images of the retinal and choroidal vasculature [2, 14, 15]. It is use in patients with ASs has shown good sensitivity in the early detection of asymptomatic, nonexudative CNV lesions before they become clinically significant, as well as in characterizing the anatomy, morphology, and vascular remodeling of the abnormal vessels conforming to the CNV after anti-VEGF therapy [3, 5, 11, 16]. These findings suggest that OCTA could be implemented as a screening tool for the early detection of disease activity, for treatment response assessment, and for the identification of early markers of recurrence.

The purpose of the present study was to report the long-term anatomical and functional outcomes, as well as the risk for recurrence of disease activity, in patients with AS-CNV treated with anti-VEGF intravitreal injections according to CNV morphology and characteristics determined by OCTA.

## Materials and methods

This was a retrospective case-control study that was approved by the internal review board of each participating hospital: Asociación para Evitar la Ceguera IAP, the Instituto Fundación Conde de Valenciana, and Hospital de Nuestra Señora de la Luz IAP. The study was conducted according to the tenets of the Declaration of Helsinki and Good Clinical Practice guidelines. All sensitive data were managed according to the Mexican Federal Law for the Protection of Personal Data in Possession of Individuals (NOM-024-SSA3-2010) and the Health Insurance Portability and Accountability Act (HIPAA) rules. Due to the retrospective nature of the study, an informed consent form was not needed. No generative artificial intelligence software was used to create or correct the text of this manuscript.

We reviewed the electronic medical records of patients aged 18 years or older with a clinical diagnosis of ASs who developed exudative or subclinical CNV between 2016 and 2022 and underwent OCTA at the time of diagnosis. All the participating patients were treatment-naïve at the moment of diagnosis, were treated with intravitreal anti-VEGF injections, regardless of the selected drug, and had a minimum documented follow-up time of 12 months. We excluded patients with a history, diagnosis, or suspicion of exudative age-related macular degeneration; myopic refractive error of -3.00 diopters or greater; occlusive retinal vascular disease; vitreoretinal pathologies; central serous chorioretinopathy; uveitis; retinal vasculitis; glaucoma; significant past ocular trauma; significant media opacities; poor pupil dilation that prevented good quality OCTA; macular fibrosis; or incomplete follow-ups/medical records. We used patients with a confirmed clinical diagnosis of myopic CNV (mCNV) as the control group, extracting their data on file.

The following data were extracted from each electronic medical record: general demographic data (age at the time of diagnosis, sex, refractive error), best corrected visual acuity at the time of diagnosis (BCVA), type of anti-VEGF drug selected for treatment, number of intravitreal injections necessary for achieving primary inactivation of the CNV, treatment regime, number of reactivations (new subretinal and/or intraretinal fluid, new retinal hemorrhages, new visual symptoms such as metamorphopsia, and newly developed visual loss of at least 5 letters or more), time (in months) between initial inactivation and disease reactivation, final BCVA on file and total time (in months) of follow-up. In the case of more than one reactivation, we extracted the time (in months) between each exudative episode, the number of needed intravitreal injections during each episode for achieving disease control, and the BCVA at the end of each new exudative episode.

AS-CNV and mCNV were diagnosed by multimodal imaging, by using the following image protocol: color fundus photography; fundus autofluorescence (DRI-OCT, Triton, Topcon Healthcare, Oakland, NJ, and Optos California, Optos, Inc., Marlborough, MA); spectral domain optical coherence tomography; and fluorescein angiography (Spectralis + HRA, Heidelberg Engineering, Heidelberg Germany).

OCTA images of both groups were obtained with an Angioplex Elite 9000 (Carl Zeiss Meditec, Inc., Dublin, USA). All enrolled patients had on file a 6 × 6 mm scan centered on the fovea. Automatic segmentation was used, provided by the Plex Elite 9000 platform, and performed segmentation of the outer retina and choriocapillaris (ORCC), during which the projection artifact removal was active. Segmentation errors were corrected manually by an experienced technician. The images were then exported and presented to the masked observers.

**Fig. 1:**
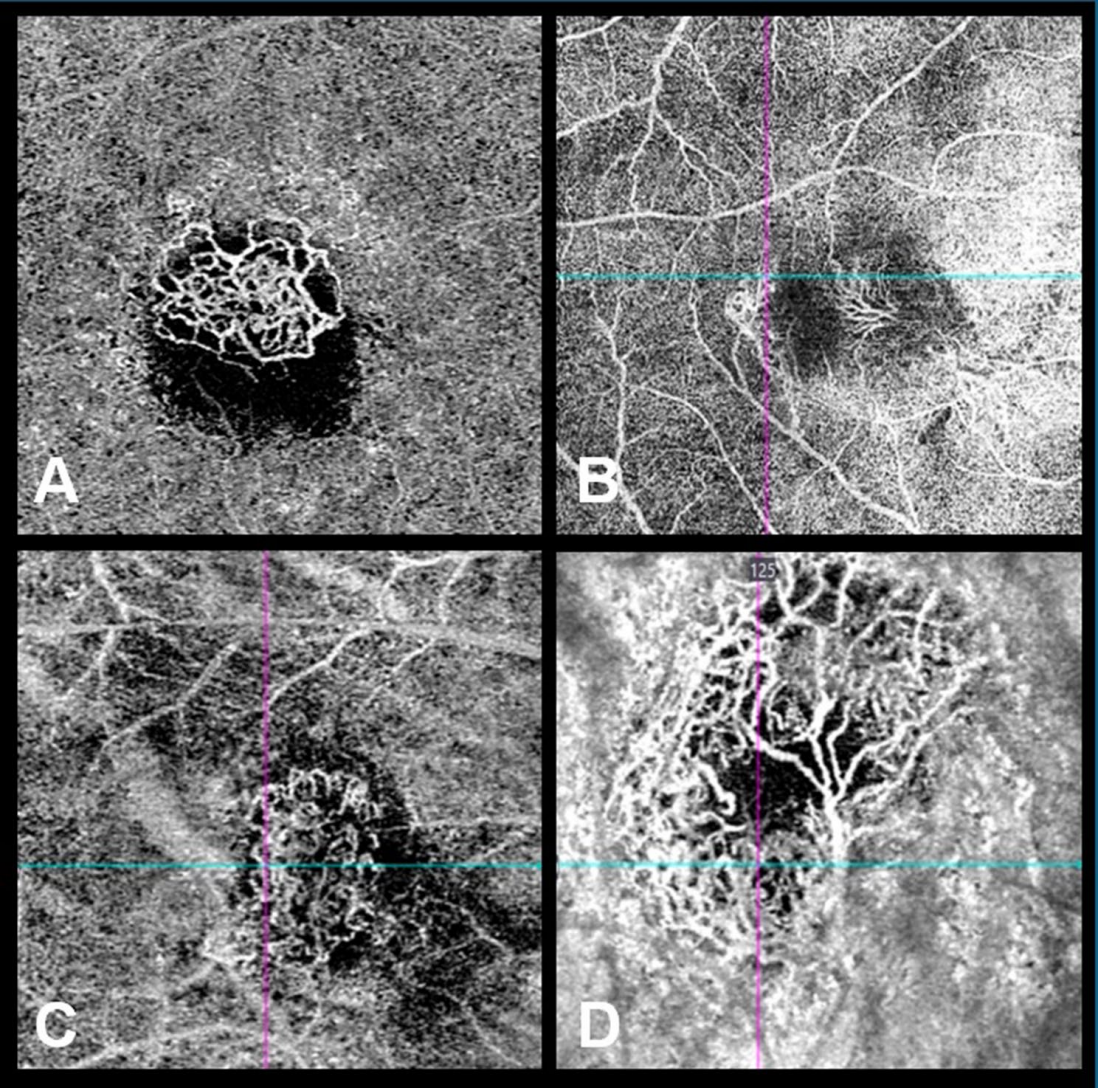
Representative optical coherence tomography angiography images of the four categories used to classify the CNV morphology at baseline. (**A**) Cogwheel (circular shape with branching and peripheral anastomosis). (**B**) Interlacing (convoluted vascular network with thin capillaries). (**C**) Mixed (combined characteristics of other CNV types). (**D**) Pruned vascular tree (filamentous flow with persistence of main vascular trunks, but no thin ramifications)

Files and image analysis of the study and control groups were performed by two independent blinded observers. Structural OCT classification of CNV and OCTA classification was performed through qualitative and morphological analysis in both groups. The structural characteristics of these patients were correlated with the risk of activity, reactivation, or poor response to treatment throughout their follow-up. On structural OCT, CNV lesions were classified as type 1 if the neovascular complex was observed between the retinal pigment epithelium (RPE) and Bruch’s membrane and as type 2 if the complex was visualized over the RPE and grew from the choroid into the subretinal space. On OCTA, CNV lesions were classified according to their morphological patterns into four categories: interlacing, cogwheel, pruned vascular tree, and mixed (Fig. 1) [17].

Statistical analysis was performed using an Excel spreadsheet (Excel 2010; Microsoft Corp. Redmond, WA) with XLSTAT v18.06 (Addinsoft, New York, NY). The general demographic data are presented as the means and proportions with standard deviation (SD) and standard error of the mean (SEM) when appropriate. The BCVA was converted into its logarithm of the minimum angle of resolution (logMAR) equivalent for statistical purposes. The visual acuity of counting fingers (CF) was 1.7 logMAR, that of hand movement (HM) was 2.0 logMAR, that of light perception (LP) was 2.3 logMAR, and that of no light perception (NLP) was 3.0 logMAR [1]. The significance of the changes in BCVA was assessed using Student’s t-test and ANOVA for repeated measurements when appropriate. An alpha value of 0.05 was considered to indicate statistical significance. Bonferroni correction was used to adjust for the significance of the alpha value. The Gaussian distribution of all variables was determined using the D’Agostino–Pearson omnibus normality test. Interobserver agreement was assessed with a Cohen-Kappa test ± confidence intervals. Odds ratios were calculated with 2 × 2 contingency tables and a Chi2 test, with an alpha value of 0.05 indicating statistical significance.

## Results

We reviewed the files of 44 patients (30 with AS-CNV and 14 with mCNV). All the files fulfilled the inclusion and exclusion criteria. The general demographic data of the study and control groups are summarized in Table 1.

**Table 1:** Demographic data, sOCT and OCTA results. Patients in the study group were significantly older than patients in the control group. There was an equal distribution of gender and affected eyes. The predominant histological type by OCT was type 2 CNV. SD: standard deviation. OD: right eye. OS: left eye. SRE: spherical refractive error. sOCT: Structural Optical Coherent Tomography. OCTA: Optical Coherent Tomography Angiography. VT: vascular tree

	Study *N* = 30 (± SD)	Control *N* = 14 (± SD)	Alpha
Age	55 ± 9.26	45.1 ± 15.2	*p* = 0.03*
Gender			*p* = 0.3
Male	15 (50%)	5 (35%)	
Female	15 (50%)	9 (65%)	
Eye			*p* = 0.3
OD	15	5	
OS	15	9	
Mean SRE (Range)	-0.06 (+ 1.5 to -2.75)	-13.75 (-3.00 to -19.75)	*p* < 0.01*
sOCT Classification			*p* = 0.9
Type 1	2 (6.7%)	1 (7.1%)	
Type 2	28 (93.3%)	13 (92.9%)	
OCTA Classification			*p* < 0.01*
Interlacing	8 (27%)	8 (57%)	
Cog-wheel	3 (10%)	5 (36%)	
Pruned VT	8 (27%)	1 (7%)	
Mixed	11 (37%)	0 (0%)	
CNV localization			
Subfoveal	53% (16)	57% (8)	
Peripapillary	37% (11)	7% (1)	
extrafoveal	10% (3)	36% (5)	

The mean baseline BCVA in the study group was 0.861 ± 0.59 logMAR. After the first round of anti-VEGF therapy, all patients in the study group were considered to be inactive due to the absence of retinal exudation. The mean BCVA improved to 0.55 ± 0.5 logMAR (*p* = 0.03). The mean number of intravitreal injections for the primary inactivation was 4.6 ± 4.3, with a 4-week interval between doses and PRN administration thereafter. The mean total follow-up time was 6.7 years (80.9 ± 138.6 months, range: 18.8 to 550 months). During the follow-up period, 33% of the patients in the study group had at least one reactivation of AS-CNV exudation, 17% had at least two reactivations, and 6% had three or more reactivations.

The mean BCVA at the time of the first reactivation of the disease in the group of affected patients was 0.471 ± 0.58 logMAR. The mean BCVA after the second round of anti-VEGF drugs was 0.715 ± 0.66 logMAR (*p* = 0.6). The mean number of intravitreal injections needed for secondary inactivation of the disease was 5.5 ± 4.9, with a 4-week interval between doses and PRN administration thereafter. The time between the primary inactivation of the disease and the occurrence of the first reactivation was 8.4 ± 6.9 months.

The mean BCVA at the time of the second reactivation of the disease in the group of affected patients exclusively was 0.473 ± 0.75 logMAR. The mean BCVA at the end of the third round of treatment with anti-VEGF drugs was 0.794 ± 0.91 logMAR (*p* = 0.4). The mean number of intravitreal injections needed for tertiary inactivation of the disease was 2.1 ± 1.0, with a 4-week interval between doses and PRN administration thereafter. The time between the first reactivation of the disease and the second was 5.8 ± 8.2 months.

**Fig. 2:**
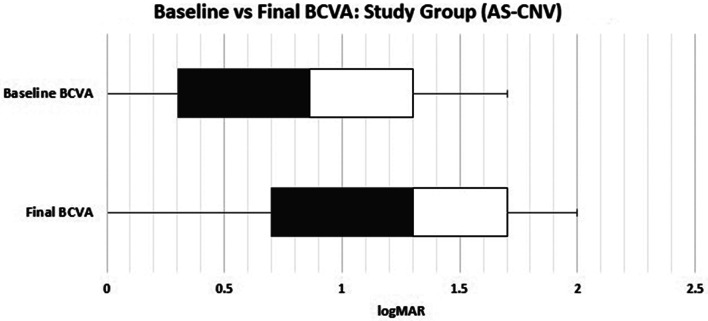
Study group visual acuity at baseline and last visual acuity recorded on file. Patients in the study group had a progressive loss of visual acuity with each episode of exudation recurrence. The last recorded visual acuity demonstrates that the initial benefit of the anti-VEGF therapy was lost

The mean BCVA at the time of the third reactivation of the AS-CNV in the affected group exclusively was 0.588 ± 0.58 logMAR. The mean BCVA after the fourth round of anti-VEGF drugs was 0.699 ± 0.001 logMAR (*p* = 0.9). The mean number of intravitreal injections needed at this time was 2.0 ± 1.6, with a 4-week interval between doses and PRN administration thereafter. The time between the second reactivation and the third reactivation was 14.7 ± 3.5 months. The mean final BCVA on file in the study group was 1.095 ± 0.61 logMAR. Figure 2 compares the BCVA at baseline and the final BCVA on file after all the study group recurrences and retreatments. The change was not statistically significant (*p* = 0.1).

In the control group, the mean baseline BCVA was 1.045 ± 0.57 logMAR. The BCVA improved to 0.501 ± 0.46 logMAR after the first round of intravitreal anti-VEGF drugs (*p* < 0.01). The mean number of intravitreal injections needed to achieve primary inactivation of the mCNV was 1.4 ± 0.8, with a 4-week interval between injections and PRN administration thereafter. The mean total follow-up time was 6.4 years (73.9 ± 12.2 months, range: 12 to 179 months). During the follow-up period, 43% of the patients in the control group had at least one reactivation of mCNV exudation, 21% had at least two reactivations, and 7% had three or more reactivations.

The mean BCVA at the time of the first reactivation of the disease exclusively in the affected patients was 0.659 ± 0.48 logMAR. After the second round of intravitreal anti-VEGF injections, the mean BCVA was 0.625 ± 0.55 logMAR (*p* = 1). The mean number of intravitreal injections needed for secondary inactivation of the mCNV was 1.8 ± 1.1, with a 4-week interval between injections and PRN administration thereafter. The time between the primary inactivation of the disease and the occurrence of the first reactivation was 9.1 ± 3.4 months.

The mean BCVA at the time of the second reactivation of the disease, exclusively in the affected patients, was 0.858 ± 0.77 logMAR. After the third round of intravitreal anti-VEGF agent injections, the mean BCVA improved slightly to 0.799 ± 0.85 logMAR (*p* = 0.8). The mean number of intravitreal injections needed for tertiary inactivation of the mCNV was 1.5 ± 0.5, with a 4-week interval between doses and PRN administration thereafter. The time between the first reactivation of the disease and the second reactivation was 11.3 ± 2.05 months.

**Fig. 3:**
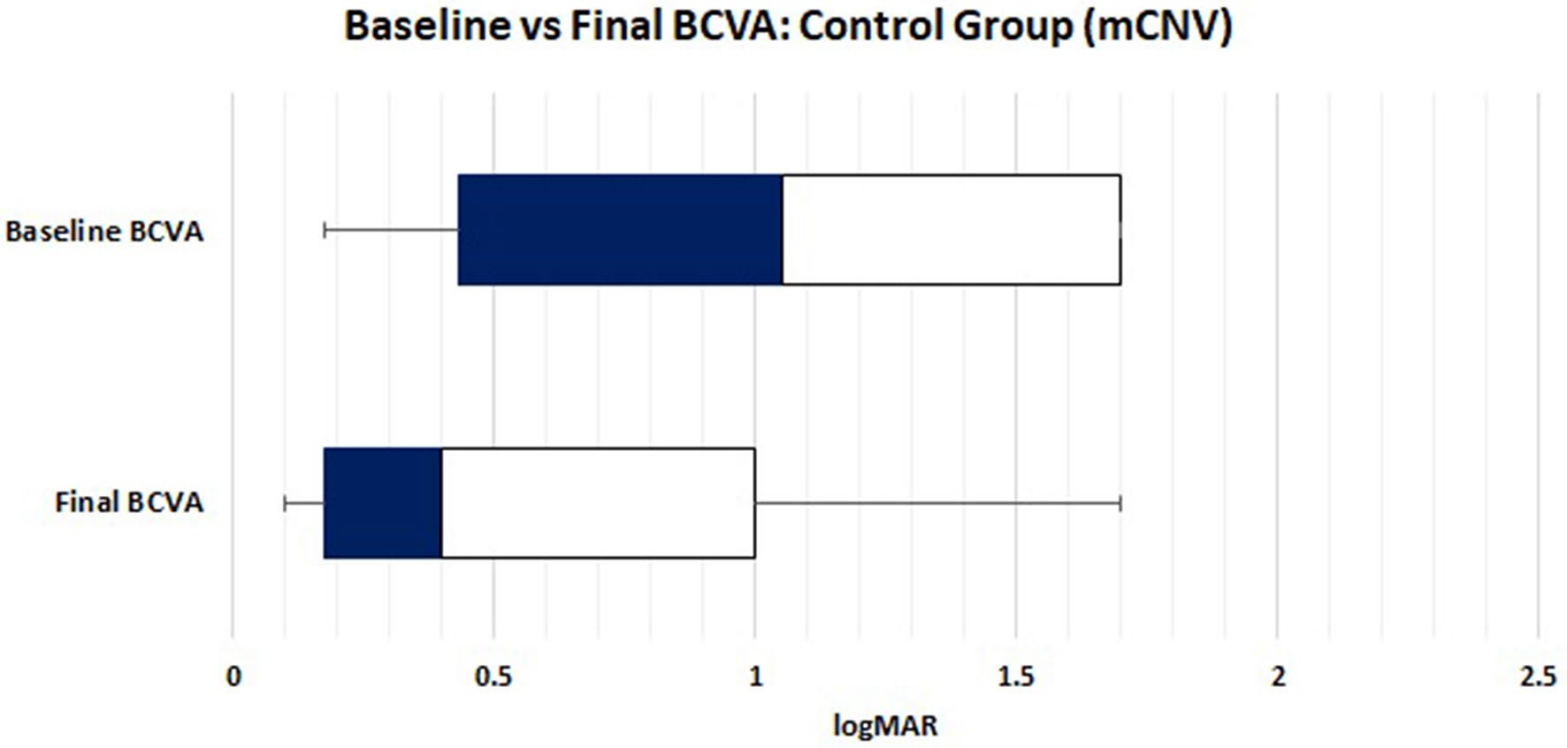
Control group visual acuity at baseline and last visual acuity recorded on file. Patients with myopic CNV improve significantly with anti-VEGF therapy. The gains were maintained through follow-up, despite episodes of exudation recurrences

Only one patient in the control group had a third reactivation of mCNV. Although his BCVA was 20/20 (0 logMAR), multimodal imaging demonstrated a new collection of intraretinal fluid (parafoveal). The patient was treated with three additional doses of intravitreal anti-VEGF drugs on a monthly basis. The patient maintained 20/20 vision, and a complete dry macula was confirmed via multimodal imaging. The patient was placed on a PRN scheme thereafter. The third reactivation occurred 4 months after the previous reactivation, and the patient’s condition remained inactive as of the last visit on file. The mean final BCVA on file in the control group was 0.617 ± 0.53 logMAR. Figure 3 compares the BCVA at baseline and the final BCVA on file after all the control group recurrences and retreatments. These changes were statistically significant (*p* = 0.05).

There was strong interobserver agreement for the structural OCT classification of CNV (Cohen-Kappa: 0.66–0.87) and for the morphological classification by OCTA (Cohen-Kappa: 0.65–0.83) in both the study and control groups. The structural OCT and OCTA classifications of the CNVs in the study and control groups are summarized in Table 1. There were no differences in structural OCT findings since the CNVs were predominantly type 2 in both groups (*p* = 0.9). The predominant pattern on OCTA in the study group was mixed, with an equal distribution of interlacing and pruned vascular tree patterns, while in the control group, the predominant patterns were interlacing and cogwheel (*p* < 0.01). The odds ratios for recurrence of exudation in the study and control groups are summarized in Table 2. In the study group, patients with mixed and cogwheel patterns had the greatest odds of having more than one recurrence during the follow-up. However, despite the distinctive trend, it failed to achieve statistical significance. In the control group, the interlacing pattern had the greatest association with one or two recurrences, but it was not statistically significant. The patient in the control group who experienced a third recurrence also had an interlacing pattern.

**Table 2:** Study group vs. Control Group odds ratio. Risk of recurrences according to qualitative analysis by OCTA. Although there was a trend of higher odd for recurrence of the mixed and cogwheel in the study group, the observed odds did not reach statistical significance. CI: confidence interval. Rec: recurrences. VT. Vascular tree

	Study group. Odds (95%CI)						Control group. Odds (95%CI)			
OCTA Patterns	Rec 1	*p*	Rec 2	*p*	Rec 3	*p*	Rec 1	*p*	Rec 2	*p*
Interlacing	0.5 (0.09–3.6)	0.5					2.0 (0.2–17.8)	0.5	2.0 (0.1–31.9)	0.6
Cog-Wheel	4.75 (0.3–60.1)	0.2	4.72 (0.2–92.9)	0.9	19 (0.6-583.4)	0.09	0.8 (0.09–7.6)	0.7	0.8 (0.05–13.6)	0.8
Pruned VT	0.5 (0.09–3.6)	0.5								
Mixed	1.2 (0.2–5.9)	0.7	7.4 (0.6–79.9)	0.09	1.8 (0.1–34.4)	0.6				

The number of intravitreal injections needed for disease inactivation was significantly greater in the study group than in the control group at all endpoints (*p* < 0.01).

## Discussion

The development of new CNV is the single most important cause of acute visual loss in patients with AS [12, 18]. Its common occurrence in young adults of working age (50 years of age or less) is even more relevant because its high incidence of recurrence, potential for scarring, and permanent visual disability may damage a patient’s employment prospects, cause significant loss of personal revenue, disrupt the patient’s family dynamics, and worsen his or her quality of life [12, 13, 19–21]. The current study describes the long-term visual outcome, response to anti-VEGF treatment, and rate of disease recurrence over time in a group of patients with AS-CNV. Moreover, the risk of recurrence was calculated according to qualitative analysis of CNV lesions by OCT and OCTA, and the findings were compared against those of a group of patients who were long believed to exhibit a similar evolution and response to treatment (mCNV).

The results reported herein confirm that although AS-CNV did have a similar appearance on structural OCT to that observed in patients with mCNV (predominantly type 2 lesions) and although patients in the mCNV group had a similar rate of disease recurrence in a shorter period of time (4 years), the visual outcome at the end of the 6-year follow-up in the AS-CNV group was significantly worse than that in the mCNV group (1.095 vs. 0.6 logMAR). Moreover, they needed, on average, significantly more anti-VEGF intravitreal injections for the inactivation of the disease (3.5 ± 1.5 vs. 1.4 ± 0.2) and for the treatment of each recurrence episode. Finally, more patients had three or more episodes of recurrence during the entire length of follow-up in the AS-CNV group than in the mCNV group. The increased number of intravitreal injections in the AS-CNV group highlights the reactive nature of the CNV in such cases, and the significant challenge of the current therapeutic strategy (PRN) is avoiding future episodes of recurrence. Therefore, we hypothesized that a more proactive approach, such as a fixed interval regime or even a treat-and-extend regimen, could potentially lead to better visual and anatomical outcomes in the long term. We currently have at our disposal longer-acting anti-VEGF agents with higher molar concentrations, such as faricimab (6 mg) and aflibercept (8 mg), which can deliver longer exudate-free periods [22, 23].

According to the OCTA findings, patients with AS-CNV had a predominance of mixed-type morphology at the time of diagnosis, while patients in the control group had more interlacing and cogwheel morphologies. Our results also showed that patients with AS-CNV with mixed and cogwheel morphologies at presentation had an increased risk for disease recurrence during follow-up (OR 1.2 and 4.75, respectively). Although our sample size is representative of a relatively uncommon disease with a substantial follow-up length, the observed patterns failed to achieve significance. In the mCNV cohort, interlacing patterns were also associated with an increased risk of disease recurrence during the follow-up period (OR 2.0), but the difference was not significant [11].

OCTA has demonstrated significant sensitivity and specificity for the early detection and characterization of CNV in numerous pathologies [24, 25]. However, the common occurrence of atrophic and fibrotic changes associated with AS-CNV progression makes the early detection of recurrences especially challenging, even when OCTA imaging is available [3, 11]. These changes could also be responsible for the apparent failure of anti-VEGF therapy in AS-CNV patients [3, 11].

In a retrospective study by Marchese et al., the authors described the 12-month follow-up of a group of 19 patients with AS-CNV [11].In addition to revealing the importance of qualitative studies of CNV lesions, the results of these studies are similar to the data reported herein. The final BCVA at the end of the 12-month follow-up in their group was very similar to that reported in our AS-CNV group after the first inactivation of the disease (0.42 ± 0.4 vs. 0.55 ± 0.5 logMAR) [11]. The same number of intravitreal injections were required to achieve this outcome (4 vs. 4.6), [11] but with the difference of having a better BCVA at baseline compared with that reported in the present study.^11^ The marked difference between the final BCVA reported by Marchese et al. and that reported by us could be explained by our significantly longer follow-up time. This allowed the atrophic and fibrotic changes associated with disease recurrence to appear. Another important difference from our study is that Marchese et al. recognized two potential markers of neovascular activity that we did not account for: the presence of vascular branching plaque and a perilesional dark halo, which were observed in 63% and 58% of their samples, respectively [11]. Another potential predictor marker for CNV activity that was unaccounted for in our baseline observations was the presence of densely packed capillary-like vessels called irregular vascular networks, as reported by Corbelli et al. and El Matri et al. [3, 26]. At present, it is not clear whether the inclusion of these three markers during our baseline observations would have increased our ability to predict recurrence. Nevertheless, in the future, it may be possible to combine such biomarkers with the observed morphology by OCTA at baseline in a mathematical index/quotient to improve the individual power of these biomarkers to predict recurrence [27].

Regarding CNV morphology determined by OCTA, Chapron et al. described the mixed type as the predominant form of CNV in their study, in concordance with our findings. [5]. Although Chapron et al. reported the interlacing pattern as more likely to be associated with disease activity and exudation [5], we believe that this does not necessarily translate to a higher risk of disease recurrence during follow-up. However, it is possible that the observation of this highly vascular morphology with straight fine vessels and no vascular loops at presentation, a morphology also observed by Falfoul et al. and Gal-Or et al. in their respective studies, could serve more as a predictor of the acute response to anti-VEGF treatment [7, 8].

Finally, in addition to its retrospective nature, our study has several limitations that we would like to acknowledge. There was a lack of treatment standardization across both groups, which suggested that the anti-VEGF agent was selected according to physician preference; all patients had individualized intervals between office visits during follow-up; general guidelines about what was considered a recurrence were followed; and the decision regarding the need for new anti-VEGF treatment was made by the physician on duty rather than by a blinded observer. A better approach could have been the standardization of the diagnosis and disease recurrence assessment by the active search for specific biomarkers associated with disease activity across all imaging modalities, such as the presence/absence of patterns of fluorescein leakage, intraretinal fluid, and subretinal hyperreflective material, among others [11]. The use of prespecified criteria for treatment and retreatment, as well as the use of a single anti-VEGF agent and identical follow-up intervals for both the study and control group. However, the authors believe that the current methodology still has value because it closely mirrors more to a real-life scenario thereby enhancing the generalizability of the results. Although each patient was instructed about signs and symptoms for early disease recurrence recognition, it is possible that some of the patients in the study group could have waited too long before seeking treatment. The ability of these patients to recognize such symptoms or slight variations in their BCVA could have also been impaired further due to recently developed scarring and atrophy throughout the follow-up. Such difficulty could also have mounted over time with each recurrence episode, negatively affecting the functional outcome of the study group. Likewise, the fact that we excluded patients with less than 12 months of follow-up possibly introduced unintended selection and survival bias. Patients with stable AS-CNV and good BCVA could have opted to not attend their corresponding follow-up visit, which pushed the mean toward a worse outcome. In contrast, patients who returned for their follow-up visit could have had more unstable disease and been more prone to recurrence and thus a negative outcome. The small sample of both groups decreases the power of the study. Particularly, the small number of patients in the control group may lead to an error type 1, which means that the odds ratio encountered here may not be representative of the pathology behavior over time. Therefore, results should be interpreted by readers with caution. Finally, although OCTA has proven to be a reliable tool for CNV detection, [17] there is still a lack of consensus regarding its qualitative assessment and the role that CNV morphological patterns could have as predictors of disease activity. Therefore, readers should consider that there might be further changes and newer CNV types, not considered at this time by the authors that could potentially change our conclusions.

## Conclusion

In summary, the current study demonstrated the natural evolution and treatment response of patients with AS-CNV. Their initial good response to anti-VEGF treatment and visual recovery is lost over time due to disease recurrence and scarring. Moreover, the long-term visual prognosis of these patients is worse than that of patients with structurally similar lesions, such as mCNV. The quantitative study of AS-CNV morphology at presentation via OCTA could help physicians predict the risk of recurrence and therefore adjust anti-VEGF treatment regimens accordingly. The authors propose that a more proactive approach to anti-VEGF treatment could lead to maintenance of the initial visual gains, less risk for scarring, and prevent deterioration of the BCVA over time

## Data Availability

No datasets were generated or analysed during the current study.

## Abbreviations

AS: Angioid Streaks
CNV: Choroidal Neovascularización;
AMD: Age-related Macular Degeneration
VEGF: Vascular Endothelial Growth Factor
AS-CNV: Angioid streaks-associated choroidal neovascularización;
PRN: Pro-Re-Nata
OCTA: Optical Coherence Tomography Angiography
HIPAA: Health Insurance Portability and Accountability Act
NOM: Normal Oficial Mexicana
IRB: Institutional Review Board
mCNV: Myopic Choroidal Neovascularization
BCVA: Best-Corrected Visual Acuity
ORCC: Outer Retina and Choriocapillaris
CF: Count Fingers
HM: Hand Movements
LP: Light Perception
NLP: No Light Perception
logMAR: logarithm of the Minimum Angle of Resolution
ANOVA: Analysis of Variance
